# Microencapsulation of Vanilla Oleoresin (*V. planifolia* Andrews) by Complex Coacervation and Spray Drying: Physicochemical and Microstructural Characterization

**DOI:** 10.3390/foods9101375

**Published:** 2020-09-27

**Authors:** Miguel Ángel Hernández-Fernández, Santiago García-Pinilla, Oswaldo Israel Ocampo-Salinas, Gustavo Fidel Gutiérrez-López, Humberto Hernández-Sánchez, Maribel Cornejo-Mazón, María de Jesús Perea-Flores, Gloria Dávila-Ortiz

**Affiliations:** 1 Departamento de Ingeniería Bioquímica, Escuela Nacional de Ciencias Biológicas, Instituto Politécnico Nacional, Carpio y Plan de Ayala, S/N Santo Tomás, Mexico City C.P. 11340, Mexico; mikess_10@hotmail.com (M.Á.H.-F.); zoidsgarcia@gmail.com (S.G.-P.); gusfgl@gmail.com (G.F.G.-L.); hhernan55@hotmail.com (H.H.-S.); 2 Facultad de Ingeniería de Alimentos, Fundación Universitaria Agraria de Colombia–Uniagraria, Calle 170 # 54a–10, Bogotá C.P. 111166, Colombia; 3 Instituto de Ciencias Básicas e Ingeniería, Universidad Autónoma del Estado de Hidalgo, Carretera Pachuca-Tulancingo km. 4.5, Carboneras Mineral de la Reforma, Hidalgo C.P. 42184, Mexico; iocampo@uaeh.edu.mx; 4 Departamento de Biofísica, Escuela Nacional de Ciencias Biológicas, Instituto Politécnico Nacional, Carpio y Plan de Ayala, S/N Santo Tomás, Mexico City C.P. 11340, Mexico; maribelpabe2@hotmail.com; 5 Centro de Nanociencias y Micro y Nanotecnologías, Instituto Politécnico Nacional, Av. Luis Enrique Erro s/n, Nueva Industrial Vallejo, Alcaldía Gustavo A. Madero, Mexico City C.P. 07738, Mexico; mpereaf@ipn.mx

**Keywords:** vanilla oleoresin, microencapsulation, complex coacervation, spray-drying, microstructure

## Abstract

Vanilla is one of the most popular species in the world. Its main compound, vanillin, is responsible for its characteristic aroma and flavor and its antioxidant and biological properties. Vanillin is very unstable in the presence of oxygen, light, and humidity, which complicates its use and preservation. Therefore, to solve this problem, this study aimed to develop vanilla oleoresin microcapsules. Vanilla oleoresin was obtained with supercritical carbon dioxide and microencapsulated by complex coacervation and subsequent spray drying (100 °C/60 °C inlet/outlet temperature). The optimal conditions for the complex coacervation process were 0.34% chitosan, 1.7% gum Arabic, 5.29 pH, and an oleoresin:wall material ratio of 1:2.5. Fourier Transform Infrared Spectroscopy (FT-IR) analysis of the coacervates before and after spray drying revealed the presence of the functional group C=N (associated with carbonyl groups of vanillin and amino groups of chitosan), indicating that microencapsulation by complex coacervation-spray drying was successful. The retention and encapsulation efficiencies were 84.89 ± 1.94% and 69.20 ± 1.79%. The microcapsules obtained from vanilla oleoresin had high vanillin concentration and the presence of other volatile compounds and essential fatty acids. All this improves the aroma and flavor of the product, increasing its consumption and application in various food matrices.

## 1. Introduction

Vanilla, one of the most important flavorings in the world, is obtained from the beans of *Vanilla planifolia* Andrews, an orchid native to Mexico [1]. Vanilla is an essential ingredient in chocolates, teas, candies, ice cream, cakes, and other products where creamy and sweet notes are required. To obtain all the aromatic compounds from vanilla beans, they undergo a treatment called curing, which includes chemical and biochemical processes and enzymatic reactions. As a result of this treatment, vanillin, the primary aromatic compound in vanilla, is released [2].

Currently, natural vanilla is marketed in four presentations: ethanolic extract, vanilla sugar, vanilla absolute, and oleoresin. Oleoresin can be obtained by extraction with ethanol, a method that was widely used but that nowadays has been changed by the supercritical carbon dioxide (SC-CO_2_) extraction since, by using this method, the problems related with alcohol residues are avoided and the method has high associated efficiencies [3,4]. SC-CO_2_ extraction is widely used in the pharmaceutical and food areas, as it has several advantages over the conventional ethanol-extraction method such as the high selectivity of the separation, reaching purities close to 100%, the products being free of pathogens, and the high efficiencies reached (1–6% *w*/*w*) [4]. Furthermore, the sensory and antioxidant properties of oleoresin are preserved, obtaining a product rich in other volatile compounds (vanillic acid and p-hydroxybenzyl alcohol) and essential fatty acids (linoleic acid and γ-linolenic acid) [5]. These advantages give the oleoresin obtained with SC-CO_2_, a unique aromatic profile compared to the other vanilla extracts [4,5].

Regarding the volatile compounds of vanilla, they are susceptible to degradation by oxygen, temperature, and humidity [6,7]. Due to this, different microencapsulation techniques have been used to preserve, store, and incorporate the aroma and flavor of vanilla in various food matrices. Among these techniques, spray drying, lyophilization, and complex coacervation stand out [8,9,10,11,12]. Complex coacervation is an optimal method for microencapsulation since no sophisticated equipment is required and high retention and encapsulation efficiencies are obtained [13]. With this technique, thermal degradation of the compounds is reduced since low temperatures are used. Another advantage is that coacervates are compatible with various systems in which their content can be released [14]. Complex coacervation is produced by electrostatic interaction between two or more oppositely charged polymers. This interaction produces two phases, a polymer-poor phase and a polymer-rich phase, which is known as a coacervate; the latter contains the active agent [15]. The most commonly used negatively charged polymers (polyanions) are polysaccharides, such as gum Arabic (GA), pectin, alginate, carrageenan, agar, and cellulose. GA is widely used due to its high solubility in water, low viscosity in solution, good surface activity, and emulsifying capacity [16,17]. Regarding the positively charged polymers (polycations), the most used ones are proteins such as gelatin, soy proteins, caseins, wheat proteins, and β-lactoglobulins [18]. The only polysaccharide considered polycation is chitosan (CH) [19,20] due to its positive ζ-potential values over almost the entire pH scale [15]. This polysaccharide is a low-cost and biodegradable compound; it has mucoadhesive properties and is non-toxic, making it attractive for applications in the food industry [9]. The combination of CH and GA results in an electrostatic complex, capable of forming strong viscoelastic films that provide a protective barrier against oxidation, light, microorganisms, and temperature [21,22]. This is because GA, compared to other polymers, has more interaction sites and negative charge for electrostatic interaction with CH [23]. Therefore, microencapsulation by complex coacervation with CH and GA is suitable. The parameters to be considered in this process are the concentration of the polymers (% *w*/*v*), pH, and temperature [24,25,26,27,28].

The application of coacervated microcapsules in various food formulations can be extended if they are dried. Lyophilization and spray drying are the most used methods for drying coacervated microcapsules [29]. Spray drying has advantages over lyophilization, such as the very short operating times, without degradation of volatile compounds in the dry product. The powders obtained have smaller particle sizes and few agglomerates. Spherical particles are obtained, which benefits the conservation and application of the powders in different products. Additionally, in most cases, spray drying is the method of choice due to its ease of scaling up and low operating costs, compared to freeze drying [30,31,32]. Moreover, an efficiency of 86.2% has been reported for the microencapsulation of natural vanilla extract using rice starch as wall material using spray drying has been reported [31]. Moreover, it has been reported that heat pumps and nitrogen can be used on yield powders obtained from vanilla ethanolic extract closer to the 80% [32].

Although there are some reports on the encapsulation of vanilla by complex coacervation [9,33], there are no studies in which the microencapsulation of complex coacervation of vanilla oleoresin from SC-CO_2_ extraction with spray-drying is reported. For this reason, this study aimed to evaluate the microencapsulation of vanilla oleoresin by complex coacervation, before and after spray drying. For the complex coacervation process, the concentration of biopolymers (CH and GA) and the pH value were optimized, considering ζ-potential, particle size, and polydispersity index (PDI) as response variables. Microencapsulation was evaluated by Fourier transform infrared microscopy (FT-IR). The microstructure of the coacervated microcapsules, before and after spray drying, was studied with environmental scanning electron microscopy (ESEM) and their chromaticity using scanning laser confocal microscopy (CLSM).

## 2. Materials and Methods

### 2.1. Materials

The cured *V. planifolia* Andrews beans from the Totonac region of Papantla de Olarte, Veracruz, Mexico were collected. The beans were stored in hermetic plastic bags (FoodSaver^®^, Oklahoma City, OK, USA) sealed by using a vacuum sealer Pack 4-2 (BBQ, USA) at 4 °C until further use. The CH (medium molecular weight, 75–85% deacetylation index) and GA used were purchased from Sigma-Aldrich (Mexico City, Mexico).

### 2.2. V. planifolia Andrews Oleoresin Extraction with SC-CO_2_

The oleoresin extraction was carried out with a supercritical fluid extraction system SFE-500 (Thar Technologies, Pittsburgh, PA, USA). The system was equipped with two pumps for solvents and two heat exchangers, one for cooling and one for heating. The temperature of the pumps and heat exchangers was regulated with a recirculating chiller containing an ethanol/water mixture. The system was also equipped with an extraction tank and a separation vessel. The oleoresin extraction was carried out at 20 MPa and 60 °C, following the methodology reported by Rojas-Ávila et al. [34]. Briefly, 100 g of the cured beans (cut into pieces of 1–25 mm thickness, 1–9 mm width, and 5–68 mm length) was placed in the extraction tank. A CO_2_ flow of 3 g min^−1^ was applied at 3 °C and 5.7 MPa for 5 h; no co-solvent was used. The SC-CO_2_, which carried the dissolved solutes, was transferred from the extraction tank to the separation vessel. During this process, the operating conditions were adjusted to 0.1 MPa and 20 °C. The dissolved solutes precipitated in the separation vessel as oleoresin, which was manually collected for use. The oleoresin was stored in an amber bottle at 4 °C until use.

### 2.3. Complex Coacervates (CCs)

#### 2.3.1. Experimental Design

A central composite design (CCD) was used to identify the configuration of the factors that optimized the response variables of the complex coacervation process. %CH, %GA, and pH were selected as factors. % CH ranged from 0.25–1.0% (*w*/*v*), % GA ranged from 1.0–5.0% (*w*/*v*), and pH ranged from 2–8 [9,24,25,26,27]. The vanilla oleoresin:wall material ratio (VO:WM) was set at 1:2.5 for all the performed runs (20). VO:WM ratio was selected according to Yang et al. [9], who used similar wall materials. The optimal CCs were obtained considering the following response variables: ζ-potential, particle size, and PDI. Numerical optimization was also applied for the modeling and prediction of the optimal complex coacervation conditions. The data were processed with Design Expert software, version 6.0.10 (Minneapolis, MN, USA).

#### 2.3.2. Preparation of the CCs

Before the formation of the CCs, two solutions were prepared, one of CH (2% *w*/*v*) in aqueous acetic acid (1% *v*/*v*) and the other of GA (10% *w*/*v*) in deionized water. Both solutions were shaken for 24 h and stored overnight at 4 °C for complete hydration. Both CH and GA were the polymers that made up the wall material (WM) of the CCs.

Initially, the vanilla oleoresin was slowly emulsified into the CH solution using an Ultra-Turrax homogenizer M45 (Ika-werke, Usingen, Germany) at 10,000 rpm for 10 min. Then, the GA solution was added to the CH with vanilla oleoresin solution and final pH was adjusted to 6.0. The mixture was stirred at 4000 rpm for 30 min and then complex coacervation was carried out by reducing the homogenization to 300 rpm and adjusting the pH of the mixture to the required value (2–8) with solutions of 0.1N HCl or 0.1N NaOH, respectively.

#### 2.3.3. Determination of ζ-Potential, Particle Size and Polydispersity Index (PDI) of CCs

ζ-potential, particle size, and PDI were determined by dynamic scattering of light (DSL), using a Zetasizer Nano ZS90 equipment (Malvern Instruments, Worcestershire, UK). For this, 10 mL of the CCs was diluted to 0.01% (*v*/*v*) using deionized water at 25 °C [24]. PDI is a measure of the width of the particle size distribution and was calculated with Equation (1) [35]:

(1) $$\mathrm{PDI}={\left(\frac{\sigma }{d}\right)}^{2}$$

where σ is the standard deviation of the diameter of the particles in the sample and d is the average hydrodynamic diameter of the particles in the sample.

### 2.4. Spray Drying of the Optimal CCs

The optimal CCs were dried in a Büchi Mini Spray Dryer B-191 (Flawil, Switzerland) with a double fluid nozzle (0.7 mm diameter). The drying conditions were 100 °C/60 °C inlet/outlet air temperature [36,37]. The solution was fed at 3.0 mL min^−1^ (10% peristaltic pump rate), and the air flow rate was 700 L h^−1^. The spray-dried microcapsules were stored in a hermetic desiccator for later characterization.

### 2.5. Characterization of the Spray-Dried Microcapsules

#### 2.5.1. Moisture Content and Water Activity (a_w_)

The moisture content of the spray-dried microcapsules was determined in triplicate using a thermobalance (Ohaus MB45, Mexico City, Mexico), according to the AOAC method 32.1.02 [38].

The *a_w_* of the spray-dried microcapsules was determined with an Aqualab water activity meter (Decagon, Pullman, WA, USA). Triplicate readings were made for each of the samples [38].

#### 2.5.2. Quantification of Surface Vanilla Oleoresin and Retention and Microencapsulation Efficiencies

Surface vanilla oleoresin (SVO) percentage and retention efficiency (RE) of the spray-dried microcapsules were determined using the methodology of Errate et al. [39]. In order to determine SVO percentage, 3 g of spray-dried microcapsules was mixed with 30 mL of isohexane. The mixture was shaken at 225 rpm for 5 min in an Orbi Shaker™ MP (Benchmark, Sayreville, NJ, USA). Subsequently, the mixture was filtered with Whatman paper (5 µm). The solid particles were washed three times with 10 mL of isohexane. The filtrate was dried with nitrogen, and the oleoresin obtained was placed in an oven at 100 °C for 1 h to remove the residual isohexane. The SVO percentage was calculated with Equation (2) [40].

(2) $$\mathrm{SVO}=\frac{W_{S}}{W_{m}}\times 100\%$$

where $W_{S}$ is the mass of the surface oleoresin and $W_{m}$ is the mass of the spray-dried particles.

RE was determined by acid digestion; 3 g of spray-dried microcapsules was mixed with 30 mL of 4N HCl. The mixture was shaken at 225 rpm for 15 min in an Orbi Shaker™ MP (Benchmark, Sayreville, NJ, USA). Subsequently, 15 mL of isohexane was added to the mixture, stirring it for 18 h at 25 °C to extract the vanilla oleoresin. Then, the mixture was centrifuged at 16,000× *g* at 20 °C for 30 min. The hexane phase, which contained the vanilla oleoresin, was dried with nitrogen and subsequently placed in an oven at 100 °C for 1 h to remove residual hexane. RE percentage was calculated with Equation (3) [40].

(3) $$\mathrm{RE}=\frac{W_{t}}{W_{m}}\times 100\%$$

where $W_{t}$ is the mass of the total oleoresin.

Encapsulation efficiency (EE) was calculated with Equation (4) [28].

(4) $$\mathrm{EE}=\frac{W_{t}-W_{S}}{W_{m}}\times 100\%$$

### 2.6. Fourier Transform Infrared Spectroscopy (FT-IR)

The analysis of the FT-IR spectrum of the CCs and the spray-dried microcapsules was performed in transmission mode with Horiba Jobin Yvon FT-IR equipment (Austin, TX, USA) coupled to a LabRam HR800 Confocal micro-Raman spectrometer (Los Angeles, CA, USA) in a spectral range of 3900 at 900 cm^−1^ with a diamond crystal and 32 scans per sample. The maximum transmittance percentage of the CCs and the spray-dried microcapsules was compared to analyze changes in the functional groups caused by spray drying [41].

### 2.7. Environmental Scanning Electron Microscopy (ESEM)

Surface morphology of the spray-dried microcapsules was observed in a 7800F environmental scanning electron microscope (Jeol Inc., Peabody, MA, USA). The following experimental conditions were used to capture the images: 2.0 kV voltage (EHT), Fil I Target LED, working distance of 10 mm, and spot size of 18 ± 0.2 µm. All micrographs were captured at 2000× and 20,000× with a size of 1280 × 960 pixels in grayscale format. The spray-dried microcapsules were mounted on cylindrical stubs which were fitted with double coated conductive carbon tape under vacuum with a 15 kV acceleration [42].

### 2.8. Confocal Scanning Laser Microscopy (CLSM)

The distribution of vanilla oleoresin in the CCs was determined before and after spray drying. CCs were stained with 0.5% oily red (St. Louis, MO, USA) (vanilla oleoresin) and 0.1% calcofluor (St. Louis, MO, USA) (wall material). Analyses were performed with an LSM 710 microscope (Carl Zeiss, Dresden, Germany) using a 63×/1.4 Oil objective. The samples were mounted on slides and observed with two lasers with 4% capacity, one laser at a wavelength of 405 nm for the fluorescence of calcofluor and the other laser at a wavelength of 633 nm for the fluorescence of oily red. The equipment detected the autofluorescence signals of the vanilla oleoresin and wall material; intensity was measured through ZEN software (from the LSM 710 microscope).

### 2.9. Chromaticity Analysis with Digital Image Analysis

The micrographs with the emission spectrum obtained by CLSM were used to determine their chromaticity and relate them to the encapsulation efficiency. The images were analyzed with the ImageJ v.1.50f software (National Institutes of Health, Bethesda, MD, USA) to obtain the RGB values and convert them into X, Y, Z (tristimulus values), thus evaluating their chromaticity coordinates *x* (R), *y* (G), and *z* (B) based on the CIE (Commission Internationale de l’Eclairage) diagram [43]. Image processing consisted of adjusting the brightness and contrast of the original image; then, the RGB channels were extracted to calculate the *x*, *y*, and *z* parameters. This system uses these three values to describe the precise location of a specific color within a three-dimensional visible color space [44]. The region of interest of each extracted channel was used to quantify the CIE parameters, x (Equation (5)), y (Equation (6)), and z (Equation (7)) [43]:

(5) $$x=\frac{X}{X+Y+Z}$$

(6) $$y=\frac{Z}{X+Y+Z}$$

(7) $$z=\frac{Z}{X+Y+Z}$$

### 2.10. Statistical Analyses

All measurements were performed in triplicate, and the mean ± standard deviation was reported. The significance of the data was evaluated by analysis of variance (ANOVA), and the comparison of means was analyzed by Tukey’s test (*p* ≤ 0.05) using Design Expert 6.0.10 software.

## 3. Results and Discussion

### 3.1. Effect of CH–GA Interaction and pH on Complex Coacervation

Table 1 shows the results obtained for ζ-potential, particle size, and PDI of the CCs, according to the complex coacervation conditions used (%CH, %GA, and pH). With these results, the optimal concentrations of the wall materials and pH values to obtain the CCs could be found. It was observed that increasing the CH:GA ratio from 1:1–1:5 with a pH of 2–5, the ζ-potential was positive (from 9.59–32.6 mV), while at CH:GA ratios >1:5 with a pH >5, the ζ-potential was negative (from −23.7 to −6.42 mV). These results were similar to those reported by Espinosa-Andrews et al. [24]; they used CH:GA ratios from 1:1–1:4.5 and obtained ζ-potentials close to 20 mV. They also reported that at CH:GA ratios >1:6.5, the ζ-potential of the CCs remained at −24.5 mV. They attributed these results to the stoichiometric relationship between the amino groups of CH and the carboxyl groups of GA. It has been reported that the lowest electrostatic attraction between CH and GA, generated by the neutralization of the electric charges of their functional groups, occurs with a CH:GA ratio of 1:5 [45]. Therefore, at CH:GA ratios >1:5, the charge balance between the molecules is lost, which decreases the performance of the CCs [26,28,46].

In all the experimental runs shown in Table 1, the CCs maintained a particle size in the range of 85.86–1936.38 nm and a PDI in the range of 0.27–0.88. Both the particle size and the PDI of the CCs were dependent on the variation of ζ-potential. The minimum particle size of the CCs (85.86 nm, run 13) was obtained with a ζ-potential of −19.23 mV and PDI of 0.39. The maximum particle size of the CCs (1936.38 nm, run 10) was obtained with a ζ-potential of 0.56 mV (the closest to neutrality among all the runs) and with a PDI of 0.27 (*R*^2^ = 0.9696). This occurred due to the charge of small particles [24] since, due to the increased pH, the NH_3_^+^ groups began to deprotonate to form –NH_2_ until reaching the isoelectric point (pKa~6.5) [47] and as the –COO^−^ group (pKa = 1.8–2.2) increased, causing the pH to increase and, consequently, the ζ-potential to decrease [22] causing to larger particle sizes. Under these conditions, the maximum interaction between CH and GA was presented, allowing the formation of the largest CCs.

Particle size and PDI are considered essential factors that influence the absorption capacity of active agents; the best values for particle size range from 45–895 nm, while PDI should be >0.5 [24]. The lowest PDI (0.27, runs 3, 10, and 16) was obtained when the CH:GA ratios were used in the range of 1:1.6–1:20. Under these conditions, the particle size varied between 123.67 and 1936.38 nm and the ζ-potential between 0.56 and 16.37 mV. The same behavior has been reported by some authors [24,45,46], with CH:GA ratios in the range of 1:1–1:19, in which they obtained particle sizes in the range of 46.9–9.913 nm. Therefore, the excess of one of the wall materials, in CH:GA ratios >1:5, tends to increase the particle size and PDI of the CCs.

The response variables for the 20 experimental runs of the CCD were analyzed by ANOVA, which indicated that the linear model (Equation (8)) was significant, with values of $R^{2}=$ 0.78 and $R_{adj}^{2}=$ 0.75.

(8) ζ=19.86+28.05CH−7.06GA−3.29pH

The model showed that CH is the variable with the most significant influence on the ζ-potential; therefore, the higher the CH concentration, the greater the ζ-potential of the CCs. On the contrary, when increasing GA concentration or pH, the CCs will present a lower ζ-potential. This phenomenon is due to the fact that CH acts as a polycation in a pH range of 2–6, causing high protonation of its functional groups (NH_3_^+^), due to the acidic conditions of the environment. Thus, by increasing pH, NH_3_^+^ groups begin to deprotonate to form –NH_2_ until reaching the isoelectric point (pKa~6.5), where CH precipitates [47]. Regarding GA, it has a negative ζ-potential in a pH range of 2–8 because its functional groups (–COO^−^) are protonated (pKa = 1.8–2.2). By increasing pH, the number of –COO^−^ groups increases, and therefore the ζ-potential decreases [22].

The linear model was optimized in order to find the CH:GA relationship and pH value that would provide a ζ-potential closest to the isoelectric point (0 mV), maximizing the size of the CCs (734.30 nm) and minimizing the PDI (0.57). This allowed obtaining stable CCs for subsequent spray drying. Table 2 shows the results of the numerical optimization of the CCD. The CCs formulation with the lowest concentration of solids (Formulation 8) was selected to be spray-dried since, with this technique, higher encapsulation efficiency has been reported when working with the smallest amount of solids [48].

### 3.2. Chemical and Microstructural Characterization of CCs and Spray-Dried Microcapsules

CCs were prepared with Formulation 8 indicated in Table 2 and with a VO:WM ratio of 1:2.5; subsequently, the CCs were spray dried. Both the CCs and the spray-dried microcapsules were chemically and microstructurally characterized to evaluate the effect of spray drying on the CCs.

#### 3.2.1. Fourier Transform Infrared Spectroscopy (FT-IR)

**Figure 1:**
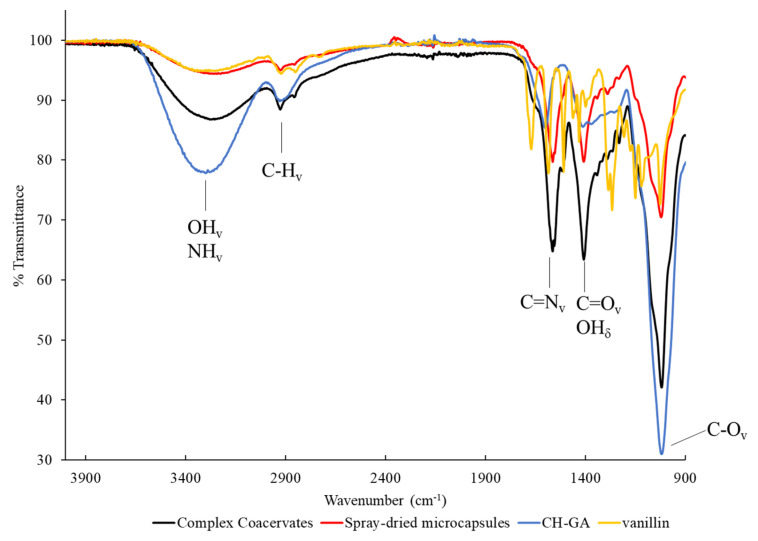
FT-IR (Fourier Transform Infrared Spectroscopy) spectra of CH-GA, vanilla oleoresin, complex coacervate and the spray-dried microcapsules. δ: bending vibrations; ν: stretching vibrations.

Figure 1 shows the FT-IR spectra of the vanilla oleoresin, wall material (CH-GA), CCs and the spray-dried microcapsules. The FT-IR spectra of vanilla oleoresin is depicted in yellow with the following bands. The broad band at 3345 cm^−1^ corresponds to the overlapping of O-H peaks belonging to water and phenols in the vanilla oleoresin. The vibrations due to stretching and bending of benzene rings. Anisaldehyde and guaiacol were in the region of 1670, 1582, 1508 cm^−1^. According to Socrates [49], the peak at 1432 cm^−1^ represents the region of O-H out of the plane deformation of phenols. While vibrations of aryl aldehydes correspond to the bands at 1264 cm^−1^. Finally, the band at 1149 cm^−1^ correspond to vibrations due to stretching and bending of methyl and ethoxy groups. In the FT-IR spectra of the CCs, a wide band was observed at 3270 cm^−1^, which is related to the –OH groups of water and –N–H groups (superimposed) of the glucosamine units of CH. The band of the C–H groups was observed at 2926 cm^−1^ and corresponds to galactose, arabinose, and rhamnose found in CH, as well as in vanillin. This band indicated the C–H stretching vibrations of the methyl and methylene groups [50].

However, the most important band was observed at 1645 cm^−1^, which is indicative of the presence of C=N functional groups [51]. These groups are produced by the nucleophilic addition of a carbonyl group to an aliphatic or aromatic amine to form hemiamines and subsequently, by dehydration, to generate imines, known as Schiff bases. Therefore, the band at 1645 cm^−1^, indicative of the C=N functional groups, allowed the confirmation of the reaction between the carbonyl groups of vanillin (C=O) and the amino groups (–NH_2_) of CH [52]. Additionally, in the 1590–1610 cm^−1^ interval, no bands related to the bending vibrations of the CH or the typical vibratory stretching of the symmetric band of the –COO^−^ functional group of the GA were observed, as reported by other authors [27,46,50]. This suggests that the electrostatic interaction of the –NH_2_ and –COO^−^ functional groups was carried out and, therefore, the formation of the CCs was achieved. Other absorption bands that were identified at 1411 cm^−1^ were the symmetrical stretching vibrations C=O and the bending vibrations –OH of the glucuronic acid from GA. Finally, the band identified at 1022 cm^−1^ represents the vibrations of the C–O groups corresponding to sugars of GA [46,53,54].

In Figure 1, when comparing each one of the FT-IR bands of the vanilla oleoresin, the biopolymers, the CCs against those of the spray-dried microcapsules, a general decrease in the % transmittance of all the bands was observed. Table 3 shows the % transmittance of the different functional groups found in the CCs and the spray-dried microcapsules. The band at 3270 cm^−1^, related to the –OH and –NH functional groups, decreased by 7.63%.

This decrease was attributed to the loss of water from the CCs during the drying process. During the spray drying of the CCs, water was transported from the interior to the surface of these by capillarity [55], where it evaporated. Subsequently, the moisture content of the already-formed spray-dried microcapsules decreased even more until reaching a state of equilibrium with the drying air. Meanwhile, after spray drying, the % transmittance of the bands at 2926, 1411, and 1022 cm^−1^ associated with the C–H_v_, C=O_v_, –OH_δ_, and C–O_v_ functional groups, decreased 6.58%, 6.12%, and 28.06%, respectively.

These functional groups (Table 3) present in the structures that formed each one of the components of the CC’s (CH, GA, and vanilla oleoresin). The decrease in the transmittance corresponding to these bands may be related to a significant change in the CCs’ rearrangements during the formation of the microcapsules. Before drying, the biopolymers (GA and CH) that formed the CC’s were highly hydrated (moisture content ~97% wet basis), so biopolymer–water interactions predominated (Figure 1 and Table 3). After drying, the water bound to the biopolymers (moisture content ~3.80% dried basis), which now made up the spray-dried microcapsules decreased, so biopolymer–biopolymer interactions predominated [55]. Finally, after spray drying, the transmittance of the band associated with the C=N functional group decreased by 14.83% which could be related to the interaction of vanilla oleoresin (carbonyl groups) and CH (amino groups) on the surface of the microcapsules (Figure 1 and Table 3). Therefore, a relevant number of the products of the interaction could have been removed after the spray drying process and directly influence the encapsulation efficiency.

#### 3.2.2. Confocal Scanning Laser Microscopy (CLSM) and Chromaticity

**Figure 2:**
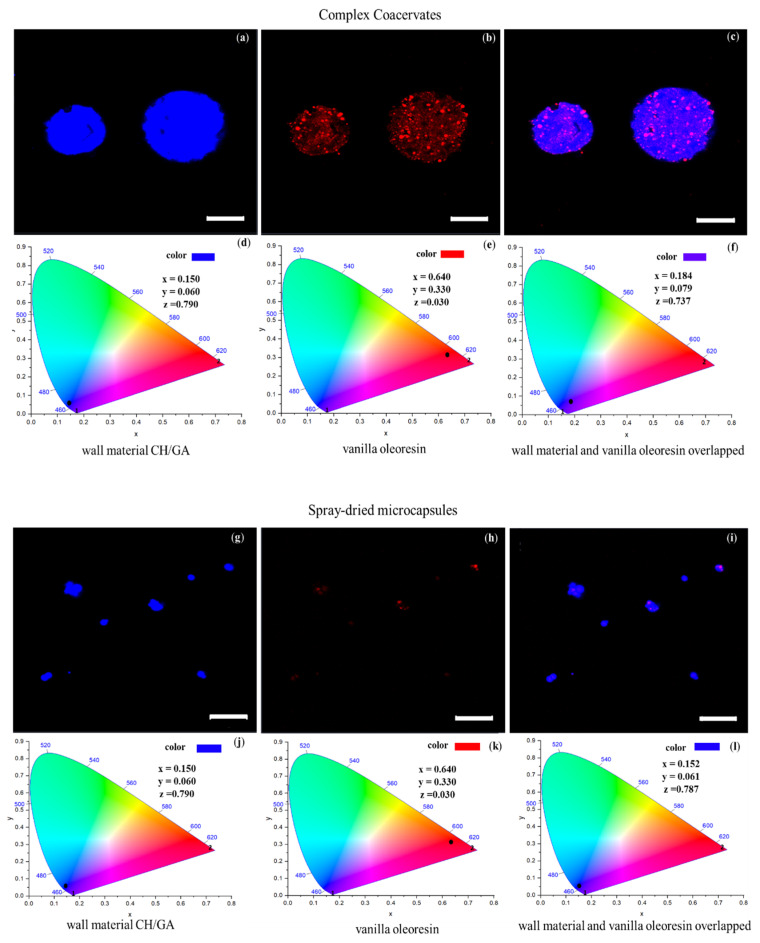
CLSM (Confocal Scanning Laser Microscopy) of complex coacervates: (**a**) wall material (CH/GA), (**b**) vanilla oleoresin, (**c**) wall material and vanilla oleoresin overlapped; CIE 1931 coordinates of complex coacervates: (**d**) wall material (CH/GA), (**e**) vanilla oleoresin, (**f**) wall material and vanilla oleoresin overlapped; CLSM of spray-dried microcapsules: (**g**) wall material (CH/GA), (**h**) vanilla oleoresin, (**i**) wall material and vanilla oleoresin overlapped; CIE 1931 coordinates of spray-dried microcapsules: (**j**) wall material (CH/GA), (**k**) vanilla oleoresin, (**l**) wall material and vanilla oleoresin overlapped. Scale bar: 10 μm.

Figure 2 shows the CLSM images and their respective CIE 1931 coordinates of the CCs and the spray-dried microcapsules. The CLSM images allowed us to observe the oleoresin distribution in the CCs and spray-dried microcapsules. Figure 2a,g shows the wall material with blue fluorescence, Figure 2b,h shows the vanilla oleoresin with red fluorescence, and Figure 2c,i shows the melting of the wall material with vanilla oleoresin with blue and red fluorescence.

The CCs, before and after spray drying, showed multinuclear encapsulation. This was probably due to the high speed of homogenization (>9000 rpm) used in the formation of this type of CCs [40]. In the literature, multi-core capsules were generally recognized as having better release control properties compared to single-core capsules. Furthermore, multi-core capsules slowly released their contents, at different discharge times; in contrast, single-core capsules released their contents in a single discharge [56]. Likewise, it has been reported that the release of flavors and aromas in mononuclear structures was less stable when exposed to heat or high concentrations of chloride salts (>100 mM). This happened because the electrostatic bonds between the biopolymers that formed the single-core capsules were broken [57].

Laser excitation of calcofluor (405 nm) and oily red (633 nm) produced specific emission spectra for the wall material and the vanilla oleoresin. These spectra were converted to *x*, *y*, and *z* color coordinates using the CIE chromaticity diagram [43]. In this way, the color produced by the wall material, the vanilla oleoresin, and the wall material–vanilla oleoresin overlap, were determined. Figure 2d,j shows the non-variation of the CIE coordinates of the wall material in the CCs and the spray-dried microcapsules. Similarly, Figure 2e,k shows the non-variation of the CIE coordinates of the vanilla oleoresin in the CCs and the spray-dried microcapsules. Figure 2f shows the effect of complex coacervation on the CIE coordinates (*x* = 0.184, *y* = 0.079, and *z* = 0.737), which produced a purple color. It was found that the increase in the *y* coordinate in Figure 2f with respect to the value of *y* in Figure 2a was associated with the interaction between the wall material (CH-GA) and the vanilla oleoresin. At the same time, this interaction was attributed to the C=N bond of the carbonyl and amino groups observed in Figure 1 [51].

Figure 2l showed that with spray drying, the overlapped CIE coordinates of the wall material and vanilla oleoresin changed from those shown in Figure 2f, giving a color transition from purple (Figure 2f) to blue (Figure 2l). The predominance of the blue color in the spray-dried microcapsules indicated a lower proportion of oleoresin retained in the wall material. This result is consistent with the C=N bond decrease mentioned in the previous section. Furthermore, the color change from purple to blue suggested higher retention and encapsulation of vanilla oleoresin by the wall materials (Table 4); these parameters were analyzed in the next section.

The CCs (Figure 2a,c) and the spray-dried microcapsules (Figure 2g,l) presented spherical morphologies. This characteristic is obtained when CH and GA are used stoichiometrically with similar charges (+/−) given by their –COO- and –NH_3_^+^ groups. This behavior has been reported by other authors using CH:GA ratios in the range of 1:2.45–1:5 (pH 3.5–5.5) [26,27,28]. In some cases, CCs did not form efficiently and instead formed thin fibrils. This is because the stoichiometric relationship between the wall components was not used. Fibrillar structures could be related to the change from coacervate to a gel phase, including a reduction in electrostatic repulsion [27]. Furthermore, if the stoichiometric ratio does not reach equilibrium, the size of the CCs tends to be small (≤1 µm) and loses sphericity, which makes it more difficult to trap the active compound. In this way, the loss of the spherical shape decreases the encapsulation efficiency of bioactive compounds, but it also limits the application of CCs in food matrices due to their large size [28]. Other investigations [58,59,60] have reported that pH, temperature, and the stoichiometric relationship were the factors that determined the interactions between charged biopolymers. Furthermore, these variables directly affect the formation of the CCs, as well as their size, stability, morphology, and physical properties. Therefore, for the protection of bioactive compounds by complex coacervation, all the variables mentioned above must be considered.

#### 3.2.3. Physicochemical Characterization of Spray-Dried Microcapsules

Table 4 shows the physicochemical properties of the spray-dried microcapsules. Its water activity (*a_w_*) was 0.13 ± 0.01, and its moisture content was 3.80 ± 0.02% dry basis. These values are characteristic of spray-dried products and are adequate to provide microbiological stability and protection against lipid oxidation [61]. The encapsulation efficiency (EE) was 69.20 ± 1.79% using a 1:2.5 VO:WM ratio. This EE is similar to those reported by Hasanvand and Rafe [33], in the range of 61–68%. They made a complex coacervation of rice bran protein, B-cyclodextrin, and vanillin, using a 1:2 active principle:wall material ratio. In contrast, Yang et al. [9] reported a higher EE (82.9%) compared to this work, using the same wall materials (CH and GA) and a 1:2 vanillin:wall material ratio. It is important to note that the mentioned study used freeze drying as a drying method. Therefore, the physicochemical properties of lyophilized microcapsules such as oxidative stability, surface oleoresin, EE, and microstructure are usually different compared to spray-dried vanillin microcapsules [9,10,11,12,33,62]. One of the main characteristics of spray-dried microcapsules is that they tend to be less porous and have a spherical shape since spray drying times are shorter compared to lyophilization.

Retention efficiency (RE) of the spray-dried microcapsules was 84.89 ± 1.94%. This physicochemical parameter is essential since it indicates the amount of total oleoresin in the microcapsules. RE was obtained by adding the encapsulated oleoresin, reported as EE (69.20 ± 1.79%), plus the non-encapsulated, reported as surface vanilla oleoresin (SVO) (15.69 ± 0.41%). The latter corresponds to the amount of oleoresin adhered to the last coating layer of the wall materials. RE, EE, and SVO are related to the morphology of the microcapsules and the adhesion forces between them [44].

#### 3.2.4. Morphology of Spray-Dried Microcapsules

**Figure 3:**
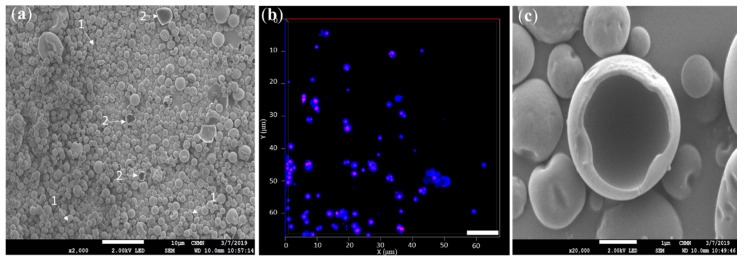
Images obtained by ESEM (Environmental Scanning Electron Microscopy) and CLSM of the spray-dried microcapsules (T_i_ = 100 °C, T_o_ = 60 °C); (**a**) ESEM image of the external structure and morphology: (1) smooth surface spherical, (2) smooth surface spherical with fracture; (**b**) CLSM image, interior: oleoresin (red), exterior: wall material (blue); (**c**) ESEM image of fractured spray-dried microcapsule. Scale bar = 10 μm.

Figure 3 shows the images of the spray-dried microcapsules obtained by SEM and CLSM, in which agglomerated microcapsules can be observed. This agglomeration was due to the non-encapsulated oleoresin that remained on the surface (SVO) and produced dynamic adhesion forces between the microcapsules [63].

As mentioned before, a contraction of the microcapsules was observed due to the loss of water vapor during spray drying (T_i_ = 100 °C, T_0_ = 60 °C) (Figure 3a,c). Figure 3a,b shows various clusters of individual microcapsules forming larger clusters, similar to that reported by Roldan-Cruz et al. [64]. Figure 3a,c shows that most of the spray-dried microcapsules had a spherical and smooth surface, while a minority of them also presented fractures. Figure 3b shows that the vanilla oleoresin (red) remained inside the microcapsules surrounded by the wall material (blue). Microcapsules with spherical and smooth surfaces are the result of shrinkage because the spray drying process is fast. Additionally, the low presence of fractured microcapsules was due to the adequate selection of wall materials that developed membranes that were permeable to water vapor, which do not fracture so easily [65,66]. However, the few fractured microcapsules (Figure 3c) appeared as a result of the formation and subsequent rupture of a non-permeable membrane, due to internal vapor pressure, which leads to the loss of vanilla oleoresin [48]. The formulation and preparation of the CCs affect the structural stability of the microcapsules. It has been reported that the formulations with higher protein content [63,64] in this case the CH with 8.5% compared to GA with 2.5% [66,67], tended to form more elastic structures, which trap a greater volume of air during homogenization or spraying. Therefore, fracture of the microcapsules could occur during the spray drying if the oleoresin does not completely occupy the interior space of the microcapsules [68,69].

## 4. Conclusions

It was possible to microencapsulate vanilla oleoresin extracted by SC-CO_2_ through complex coacervation and spray-drying microencapsulation which allowed the size of the microcapsules to be controlled, as well as the protection of the volatile compounds to be increased, producing a higher stability. The ζ-potential of the CCs, the concentration of the biopolymers (CH and GA), and the pH conditions significantly influenced the electrostatic interactions between the functional groups -NH^3+^ and COO^−^. The optimal conditions found were 0.34% and 1.7% of CH and GA, respectively, at pH 5.29 with a ζ-potential of −9.64 × 10^−5^ which allowed the performance of the complex coacervation. Spray drying of the CCs did not affect the vanilla oleoresin compounds obtaining microcapsules with an appropriate encapsulation and retention efficiencies. The chromaticity analysis revealed that the surface surrounding the vanilla oleoresin was mainly composed of wall material which indicated a protection of the encapsulated material. After spray-drying, most of the obtained microcapsules showed spherical and smooth surface morphologies. These findings could facilitate their application in different food matrices, with the added value of a higher concentration of vanillin, diversity of volatile compounds and the presence of essential fatty acids. These properties could increase and enhance the consumption of vanilla flavor using lower amounts of microcapsules.

## Figures and Tables

**Table 1:** Experimental CCD and results obtained for ζ-potential, particle size, and PDI.

Run		Complex Coacervation Conditions			Results		
	%CH + %GA		CH:GA ^1^	pH	ζ-Potential (mV)	Particle Size (nm)	PDI
1	0.63	3.00	1:4.8	5.00	−6.63	1635.33	0.88
2	0.25	5.00	1:20	8.00	−23.70	299.77	0.43
3	0.63	1.00	1:1.6	5.00	16.37	123.67	0.27
4	0.63	3.00	1:4.8	2.00	12.17	118.10	0.42
5	0.63	3.00	1:4.8	5.00	−6.91	1635.33	0.88
6	0.63	3.00	1:4.8	5.00	−6.42	1635.33	0.88
7	1.00	3.00	1:3	5.00	18.80	309.70	0.45
8	0.25	1.00	1:4	8.00	−3.56	1591.67	0.62
9	0.63	3.00	1:4.8	5.00	−6.65	1635.33	0.88
10	0.25	5.00	1:20	2.00	0.56	1936.38	0.27
11	1.00	5.00	1:5	8.00	−15.83	361.60	0.46
12	0.63	3.00	1:4.8	5.00	−7.82	1635.33	0.88
13	0.63	5.00	1:8	5.00	−19.23	85.86	0.39
14	1.00	1.00	1:1	8.00	32.60	255.43	0.42
15	0.63	2.50	1:4	8.00	−13.43	442.17	0.57
16	0.25	1.00	1:4	2.00	19.70	136.00	0.27
17	0.63	3.00	1:4.8	5.00	−6.63	1635.33	0.88
18	1.00	5.00	1:5	2.00	9.59	527.07	0.56
19	0.25	3.00	1:12	5.00	−23.77	359.93	0.51
20	1.00	1.00	1:1	2.00	29.25	226.60	0.44

CCD: Central composite design, PDI: Polidispersity Index, CH: Chitosan, GA: Gum Arabic. ^1^ The CH:GA ratio was included as an equivalent of the percentages used for each polymer, to facilitate the discussion of the obtained results.

**Table 2:** Numerical optimization of the CCD to formulate complex coacervates CCs.

Formulation	%CH	%GA	CH:GA Ratio	pH	ζ-Potential (mV)
1	0.9	3.52	1:4	6.11	−2.93 × 10^−5^
2	0.83	3.74	1:4.5	5.1	−2.96 × 10^−5^
3	0.85	3.46	1:4	5.83	−4.95 × 10^−5^
4	0.85	4.29	1:5	4.06	−5.42 × 10^−5^
5	0.7	3.39	1:4.8	4.69	8.44 × 10^−5^
6	0.8	4.01	1:5	4.24	−8.44 × 10^−5^
7	0.47	2.36	1:5	5.02	−9.59 × 10^−5^
8	0.34	1.7	1:5	5.29	−9.64 × 10^−5^
9	0.81	3.18	1:4	6.1	1.33 × 10^−5^
10	0.55	1.78	1:3	6.89	−1.26 × 10^−5^

**Table 3:** Functional groups present in the vanilla oleoresin, biopolymers, complex coacervates, spray-dried microcapsules and the difference between the CCs and the microcapsules.

Bond	–OH_v_, –NH_v_	C–H_v_	C=N_v_	C=O_v_, –OH_δ_	C–O_v_
Wavenumber (cm^−1^)	3270	2926	1568	1411	1022
% Transmittance vanilla oleoresin	94.90	94.63	77.76	83.03	72.55
% Transmittance CH-GA	77.95	89.86	88.90	86.07	31.16
% Transmittance Complex Coacervates	86.79	88.58	65.36	63.86	42.43
% Transmittance Spray-Dried Microcapsules	94.42	95.16	80.19	79.98	70.49
% Difference CCs and the Microcapsules	7.63	6.58	14.83	16.12	28.06

δ: bending vibrations, ν: stretching vibrations.

**Table 4:** Physicochemical properties of spray-dried (T_i_ = 100 °C, T_0_ = 60 °C) microcapsules.

VO:WM	*a_w_*	%Moisture	%RE	%SVO	%EE
1:2.5	0.13 ± 0.01	3.80 ± 0.02	84.89 ± 1.94	15.69 ± 0.41	69.20 ± 1.79

Data are expressed as mean ± standard deviation, *n* = 3, *a_w_*: water activity, RE: Retention Efficiency, SVO: Surface Vanilla Oleoresin Percentage, EE: Encapsulation Efficiency.
